# Evaluation of Radiation Resistance of Polystyrene Using Molecular Dynamics Simulation

**DOI:** 10.3390/ma15010346

**Published:** 2022-01-04

**Authors:** Yeong-Heum Yeon, Ha-Eun Shim, Jin-Hyung Park, Nam-Ho Lee, Jae-Yeon Park, Moon-Sik Chae, Jung-Ho Mun, Jae-Hyun Lee, Hui-Jeong Gwon

**Affiliations:** Advanced Radiation Technology Institute, Korea Atomic Energy Research Institute, 29 Geumgu-Gil, Jeongeup-Si 56212, Korea; yhyeon@kaeri.re.kr (Y.-H.Y.); she0805@kaeri.re.kr (H.-E.S.); jhpak@kaeri.re.kr (J.-H.P.); nhlee@kaeri.re.kr (N.-H.L.); jaeyeon@kaeri.re.kr (J.-Y.P.); cmswill@kaeri.re.kr (M.-S.C.); jhmun@kaeri.re.kr (J.-H.M.); jaehyunlee@kaeri.re.kr (J.-H.L.)

**Keywords:** gel permeation chromatography, scission, polystyrene, fluorinated polystyrene, molecular dynamics simulation, radiation resistance

## Abstract

The scission rates of polystyrene and fluorinated polystyrene irradiated in an irradiation facility with Co-60 γ-rays were determined using molecular dynamics simulation and gel permeation chromatography (GPC) molecular weight distributions. The prediction was based on the assumption that γ-ray energy is transferred to the initial velocity of the primary knock-on atom. We employed a molecular dynamics simulation procedure to compute the changes in bond length between the connections for selected values of the absorbed dose and compared the calculated values with measurements made on the irradiated samples. The samples were exposed to four different absorbed doses of 25, 50, 75, and 100 kGy. The scission process and scission ratio were simulated with LAMMPS with ReaxFF potential for each bond, and we compared the simulation results with the experimental data especially measuring average molecular weight to evaluate the effect of fluorination on radiation enhancement.

## 1. Introduction

Polystyrene is a standard plastic used in a radiative environment for applications in the nuclear industry and nuclear reactors and outer space localization. Irradiation experiments have been used to confirm whether these plastics can be used as phantom materials to measure absorbed doses. These materials have been increasingly used in various radiation dosimetry protocols. Solid plastics, such as polystyrene, were used for low-energy radiation dosimetry by the IAEA (2000) International Code of Practice TRS-398 [1]. The mechanical properties of these materials were measured by increasing the radiation dose to evaluate these materials. Crosslinking polymers, sterilizing, and preserving medical equipment or food are the application of radiation modification as the result of this method. These polymers have been modified to improve their radiation enhancement properties through experimental trial and error, but this method takes a long time [2,3]. Many experimental methods have been studied to increase the radiation enhancement of polystyrene by fluorination [4,5] They are comparing zero-strength-time and tensile-strength mechanically, and measuring G-value, detecting free radicals by electron spin resonance chemically [6,7].

The irradiation resistance assessment of polymers has been comprehensively performed mechanically, including tensile strength and elongation at break, for tensile tests. Several studies have shown that the “equal dose–equal damage” concept is not appropriate because of the complexity of radiation reactions in polymers [8,9]. Polymerizing, grafting, chain scission, and crosslinking are the main reactions to irradiation. The study and simulation of irradiation effects focused on crosslinking because of its importance, particularly the improvement of mechanical and thermal properties of the polymer [10,11]. The types of irradiation that cause breaking chains are electrons, neutrons, α- and β-particles, or γ- and X-rays. The chemical and physical aging of polystyrene due to the γ–ray was studied, especially the effect of air on the radiation-induced changes in mechanical and molecular properties by considering scission and crosslinking yields, G(S) and G(X) [12,13]. The crosslinking reaction in polystyrene with γ-ray was studied especially deuterated styrene was studied [14]. However, crosslinking occurs by breaking the chains to produce radicals. These radicals are the main factors involved in the crosslinking of polymers. Therefore, we simulated microscopic information obtained by theoretical methods and molecular dynamics to predict the scission ratio of each bond in polystyrene and fluorinated polystyrene and compared the simulation data with experimental data especially measuring average molecular weight to evaluate the effect of fluorination on radiation enhancement.

In this study, we simulated the scission rates of polystyrene and fluorinated polystyrene and irradiated them in an irradiation facility with Co-60 γ-rays and compared the simulation results and average molecular weight measurement. Molecular dynamics methods calculated the polystyrene and fluorinated polystyrene bond lengths by increasing the absorbed dose to predict the scission rate and provide a radiation enhancement method for each polymer. The radiation resistance was investigated by the partial substitution of H atoms for F atoms.

## 2. Materials and Methods

### 2.1. Materials

The styrene monomer, 2,3,4,5,6-pentafluorostyrene, tetrahydrofuran and di-tert-butyl peroxide (DTBP) as an initiator for polymerization were purchased from Sigma-Aldrich Korea (Seoul, Republic of Korea). 4-fluorostyrene was purchased from Tokyo Chemical Industry Co., Ltd. (Tokyo, Japan). All commercially available reagents were used as received without further purification.

### 2.2. Determination of Average Molecular Weight Sample Preparation and γ Radiation Experiments

**Figure 1:**
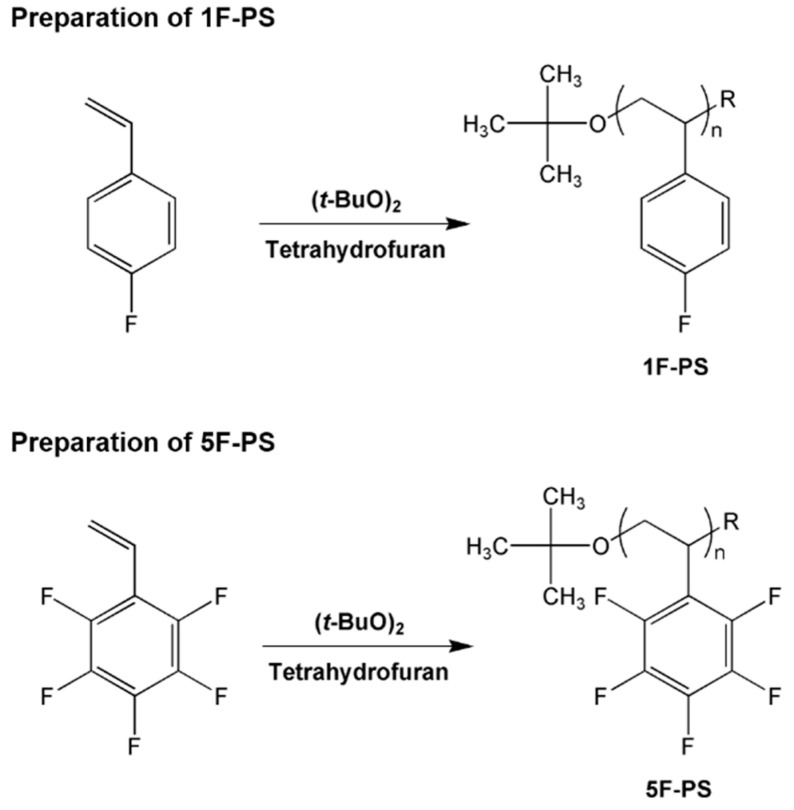
Preparation of Ps, 1F-PS(poly(4-fluorostyrene)) and 5F-PS(poly(2,3,4,5,6-pentafluorostyrene)).

Polystyrene (PS) and fluorinated styrene (1F-, 5F-PS) were synthesized as per a procedure described in the literature with slight modifications (Figure 1) [15]. The styrene monomer (10.5 g, 0.1 mol) was suspended in 10 mL of tetrahydrofuran (THF) and 1.42 g (0.01 mol) of di-tert-butyl peroxide (DTBP) was added. The polymerization was carried out at 100 °C for 18 h, at which point the solution is cooled to room temperature (20 °C). The reaction product (PS) was precipitated in a solution of methanol (200 mL), vacuum-filtered, and dried at 60 °C for five days to evaporate the remaining solvent. To synthesize the fluorinated styrene, the same procedure as described above was used with 12.2 g (0.1 mol) of unit 4-fluorostyrene to the 1F-PS and 9.7 g (0.05 mol) of unit 2,3,4,5,6-pentfluorostyrene to the 5F-PS, respectively.

Prior to gamma irradiation, the prepared samples were packed in glass vials. After that, gamma irradiation was performed with respective irradiation doses (25, 50, 75, and 100 kGy) at a dose rate of 10 kGy/h. The Co-60 source (MDS Nordion, Ottawa, Ontario Canada, IR 221 n wet storage type C-188) was located at the Korea Atomic Energy Research Institute (KAERI), Jeongeup-si, Korea.

### 2.3. Determination of Average Molecular Weight

Changes in the molecular weights of the irradiated PS, 1F-PS, and 5F-PS were determined by gel permeation chromatography (GPC, Breeze system, Waters, Milford, USA) with the eluent of CHCl_3_ at a flow rate of 1.0 mL/min at 40 °C. This equipment consisted of a Water 1515 Isocrylic HPLC pump, Water 2414 reflective index detector, Phenogel column 5 μm (300 × (4.6 mm)), and calibrated with polystyrene standards (Shodex, SM-105, and SL-105).

## 3. Theoretical Study of Scissionning Reactions

### 3.1. Modeling

**Figure 2:**
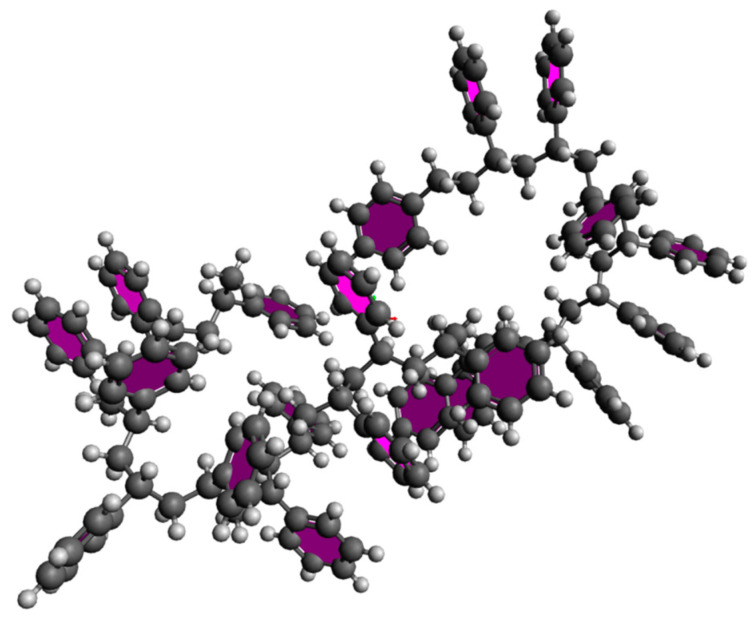
Polystyrene with 322 atoms.

The structure of polystyrene and fluorinated polystyrene were drawn using Avogadro and optimized using LAMMPS [16,17]. We applied the reactive hydrocarbon potential AIREBO and optimized its structure at room temperature [18]. The simulation size was 40 × 40 × 40 cubic Å for one cell. The polystyrene in on cell was shown in Figure 2.

**Figure 3:**
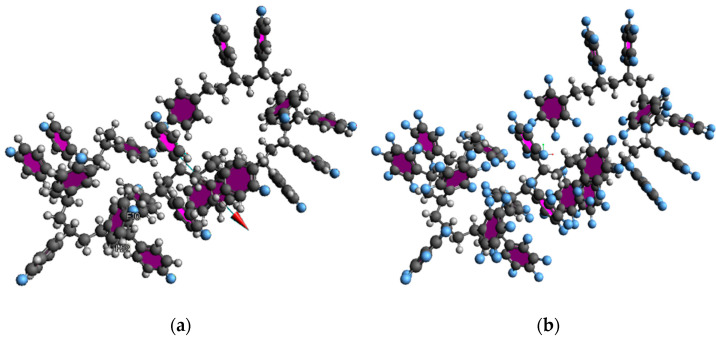
Fluorinated polystyrene: (**a**) 1F-PS(poly(4-fluorostyrene)) (**b**) 5F-PS(poly(2,3,4,5,6-pentafluorostyrene)).

We applied a reactive force field potential to consider the fluorine atoms [19,20]. The fluorinated polystyrene, 1F-PS(poly(4-fluorostyrene) and 5F-PS(poly(2,3,4,5,6-pentafluorostyrene)), are shown in Figure 3. The length of each atom was calculated and compared with the cut-off distance of each bond using the same method as that for polystyrene. We performed the simulation ten times in 10 cells under the same conditions.

#### 3.1.1. Polystyrene

For polystyrene simulation, 322 atoms in 1 cell (total 10 cells) were drawn using Avogadro and are shown in Figure 2. We optimized the atoms in Avogadro, and converted the modeling file to the LAMMPS data file.

#### 3.1.2. Fluorinated Polystyrene

For the fluorinated polystyrene simulation, we substituted the benzene ring of polystyrene with fluorine to evaluate the radiation enhancement. We optimized the atoms in Avogadro with the UFF force field and converted the modeling to the LAMMPS data file.

### 3.2. Reactive Molecular Dynamics Simulation

The scission rate was calculated using molecular dynamics simulations to evaluate the radiation resistance of polystyrene and fluorinated polystyrene. These materials were simulated using the LAMMPS code. We applied the reactive hydrocarbon potential AIREBO developed by Brenner for polystyrene and reactive force field potential (ReaxFF) developed by Van Duin et al. for fluorinated polystyrene [18,19].

High-energy particle irradiation has often been simulated using molecular dynamics codes such as LAMMPS and PARCAS, for instance, in the study of Beardmore et al. [20,21]. The high-energy particle irradiation to material is simulated by applying the interaction between the particles, the colliding electron, and the lattice atom. This method was studied by giving initial kinetic energy, the recoil energy, to some randomly chosen atom in the lattice [22,23,24].

However, this study simulated the interaction between γ-rays and atoms by providing γ-ray energy in a specific area of the chain of polymers. We converted the γ-ray energy to the velocity of the primary knock-on atom (PKA) that existed in a randomly chosen area (included recoil atom) and calculated the scission rate of each bond. This approach is suitable for investigating the scission rate of each bond during initial energy conversion.

The energy of the γ-ray was 1332 keV, and we converted it to a momentum term to calculate the velocity of the initial primary knock-on atom. The momentum of the initial primary knock on the atom was calculated using the following equation:

(1) $$P=\frac{E}{c}$$

where *c* denotes the velocity of light. The initial velocity was calculated by the equation:

(2) $$P=mv,\;\;\;v=\frac{E}{mc}$$

where *m* is the mass of the initial primary knock-on atom. We assumed that all of the γ-ray energy converted to the kinetic energy of the primary knock-on atom. The calculated velocity was 274 Å/ps for the carbon atom. The direction of the momentum was the same direction as γ-ray.

**Figure 4:**
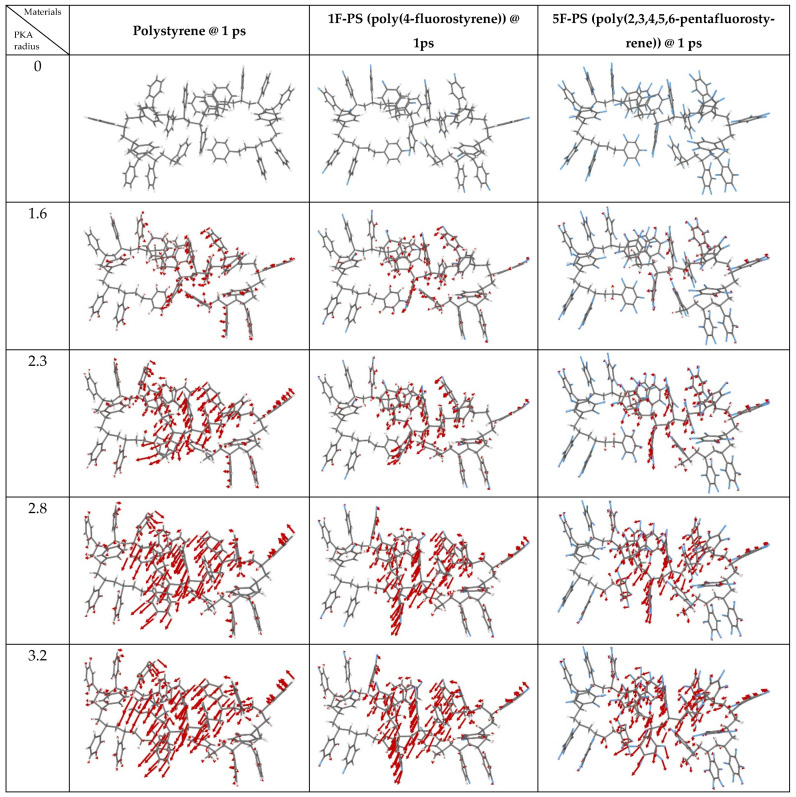
Snapshots of the displaced atoms’ velocity vector (conversion of γ-ray energy to kinetic energy-collision in the cascade zone) at the polymers @ 1 ps with increasing the PKA radius.

The second assumption was that we selected the shape of the applied velocity area sphere where collision cascade occurs. The radius of the area was 1.6 Å, which is the C-C bond length. This area was increased by increasing the absorbed dose. We applied this assumption by the equation:

(3) $$D_{n}=\frac{N_{d}}{N}=\int_{0}^{t}\int_{0}^{\infty }\int_{E_{d}}^{T_{max}}\emptyset \left(E,t\right)\frac{d\sigma \left(E,T\right)}{dt}\gamma \left(T\right)\mathrm{dTdEdt}$$

where *D_n_* denotes the probability of displacement atoms, *N* denotes number density of the material, *N_d_* denotes the number of displaced atoms per unit volume, *t* denotes irradiation time, ∅(*E*,*t*) denotes flux of incident particle, *σ*(*E*,*t*) denotes scattering cross-section, γ(*T*) denotes the number of atoms displaced from origin position by PKA, *E* denotes the initial energy of the incident particles, *T* denotes transferred energy, *E_d_* denotes the energy of the displaced atom and *T_max_* is maximum energy transferred to PKA. In the simulation, the initial energy of the incident particle (*E*) was fixed by the value of conversion energy of γ-ray as the first assumption because the radiation intensity was set by the energy of γ-ray (1332 keV). The flux of incident particle was increased proportionally to the absorbed dose, so we increased the PKA region (collision cascade zone) proportional to the absorbed dose. Because increasing absorbed dose means that increasing the number of atoms displaced from origin position (γ(*T*)) is proportional to the flux of incident particles, including the irradiation time. The number of atoms displaced from the original position and their velocity by PKA are shown in Figure 4.

The absorbed dose of polystyrene and fluorinated polystyrene was 25, 50, 75, 100 kGy in the experiment. We increased the radius of the PKA area proportional to the absorbed dose, and they were 1.6, 2.3, 2.8, and 3.2 Å in the simulation. We optimized the geometry of each material, and the simulation time was one ps. We checked the distance of each atom at 10 cells after the 1 ps-the collision process and compared them with the bond break length (1.8, 1.09, and 1.35 Å for C-C, C-H, and C-F bond) to obtain the scission rate for each bond.

## 4. Results

### 4.1. Simulation Results

The bond break lengths of each bond are 1.8, 1.09, 1.35 Å, corresponding to C-C, C-H, and C-F bonds, respectively. We obtained the distance of each bond at 1 ps after applying the velocity, which is shown in Figure 4. The maximum number of bond dissociations (@ 1 ps) for polystyrene and fluorinated polystyrene are listed in Table 1, Table 2 and Table 3.

The dissociation rate was calculated at the maximum dissociation number. The maximum dissociation rate region was 3 (#/kGy) from 25 to 50 kGy at polystyrene, 1.96 (#/kGy) from 25 to 50 kGy at the 1F-PS (poly(4-fluorostyrene)), and 2.84 (#/kGy) from 50 to 75 kGy at the 5F-PS (poly(2,3,4,5,6-pentafluorostyrene)).

**Figure 5:**
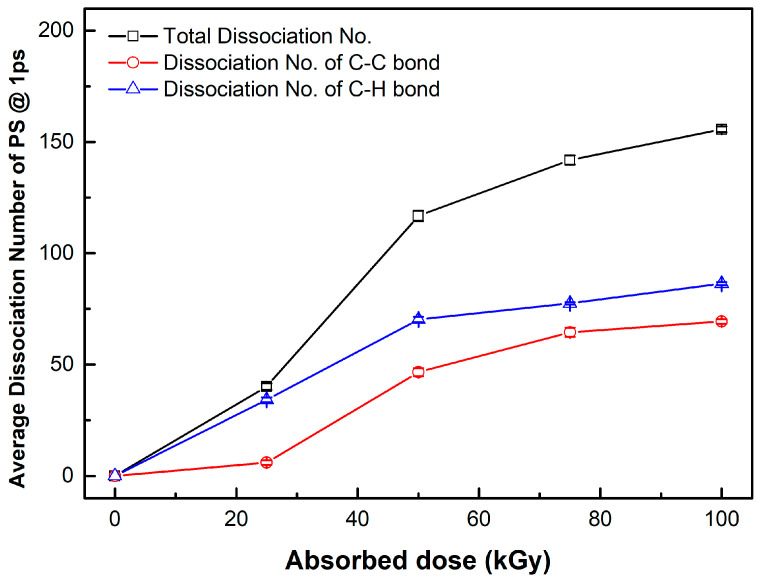
Average dissociation number of each bond in polystyrene at 1 ps.

The average dissociation number of each bond at 1 ps in polystyrene for 10 simulations is shown in Figure 5. The dissociation of C-C bonds is less than that of C-H bonds in polystyrene, implying that the scission rate of the backbone in polystyrene is less than that of other bonds.

**Figure 6:**
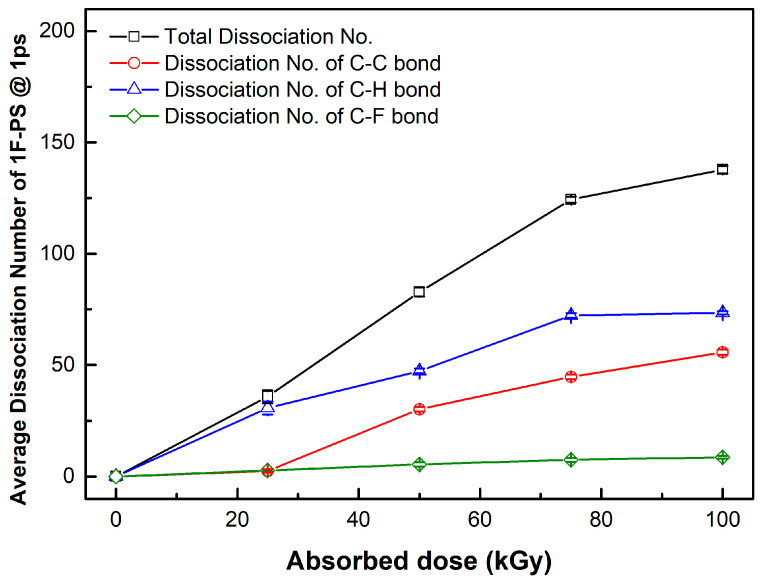
Average dissociation number of each bond in 1F-PS (poly(4-fluorostyrene)) at 1 ps.

In 1F-PS (poly(4-fluorostyrene)), the dissociation number of C-C bonds is also less than that of C-H and C-F bonds. The dissociation of C-F bonds occurred, as shown in Figure 6.

**Figure 7:**
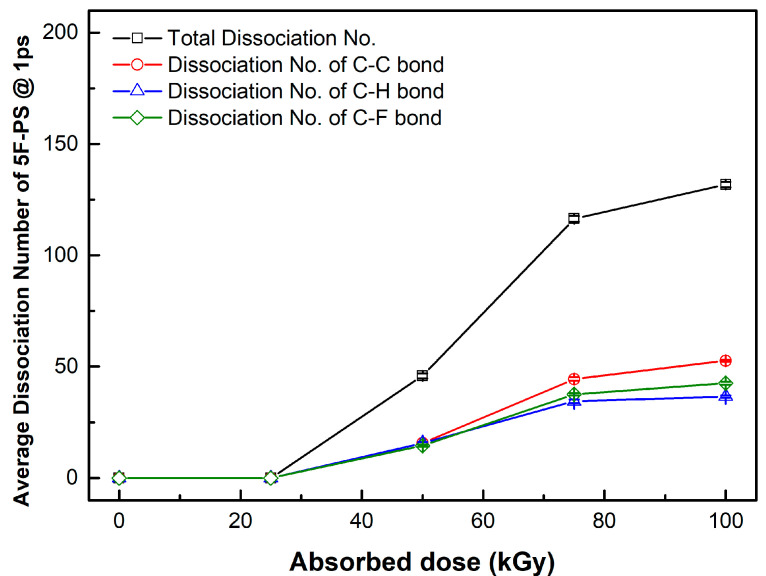
Average dissociation number of 5F-PS (poly(2,3,4,5,6-pentafluorostyrene)) at 1 ps.

The average dissociation number of each bond at 1 ps in 5F-PS (poly(2,3,4,5,6-pentafluorostyrene)) is shown in Figure 7. Dissociation did not occur up to 25 kGy. The dissociation number of C-C bonds was higher than that of C-F and C-H bonds.

**Figure 8:**
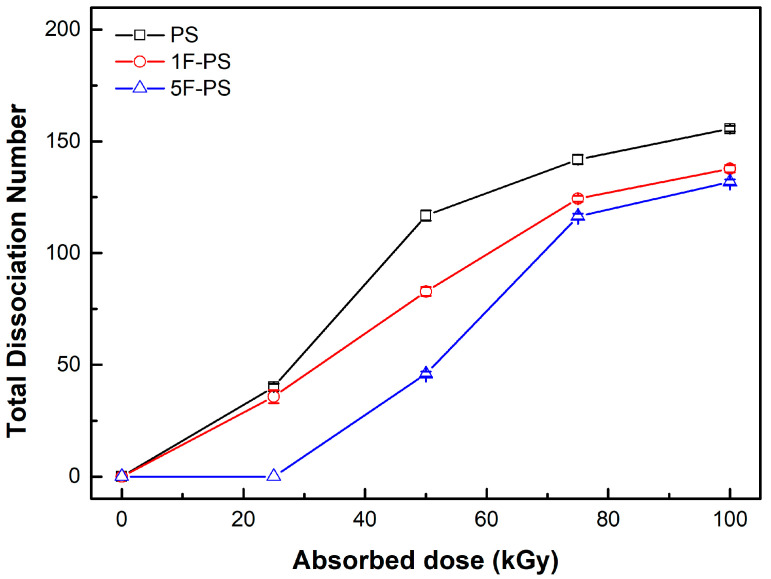
Total dissociation number of polymers.

We did not compare the dissociation number of each bond in the polymers because the total number of C-H and C-F bonds are different for each polymer. However, the total dissociation number was comparable for radiation resistance according to the same atom number. The 5F-PS (poly(2,3,4,5,6-pentafluorostyrene)) had the lowest dissociation number, as shown in Figure 8.

### 4.2. Comparison with Experiment Results

**Figure 9:**
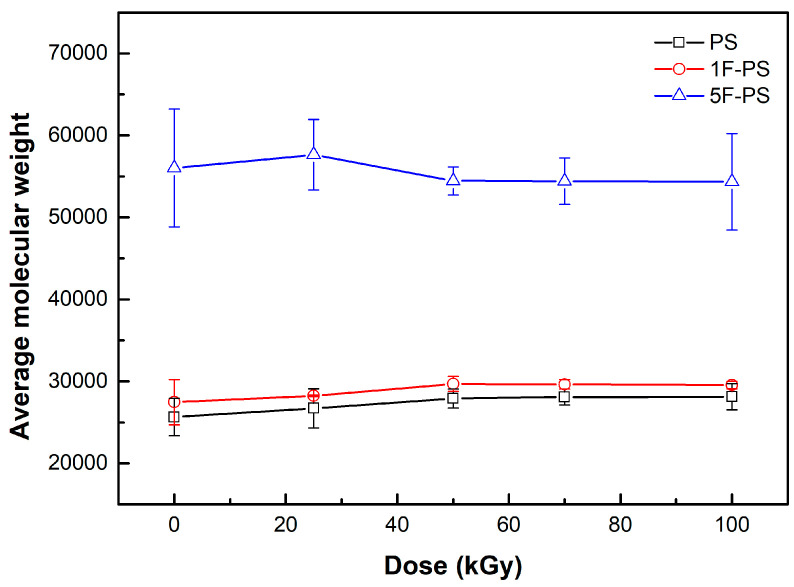
The polymers average molecular weight after irradiation (experiment results).

We applied the scission rate by considering the mass of each atom for comparison with the gel permeation chromatography results. The results of polymers’ average molecular weight (Mn) are shown in Figure 9, and the ratio of differential average molecular weight (Mn) of the irradiated polymers are summarized in Table 4. The values of Mns were different for each polymer. We applied the ratio of changes to compare the radiation resistance of each polymer. The ratio was calculated by standardizing the initial Mns value.

**Figure 10:**
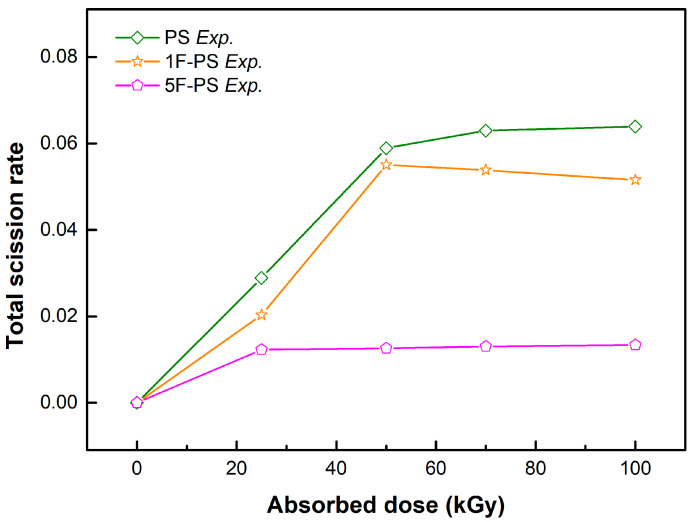
The polymers normalized average molecular weight after irradiation (experiment results).

**Figure 11:**
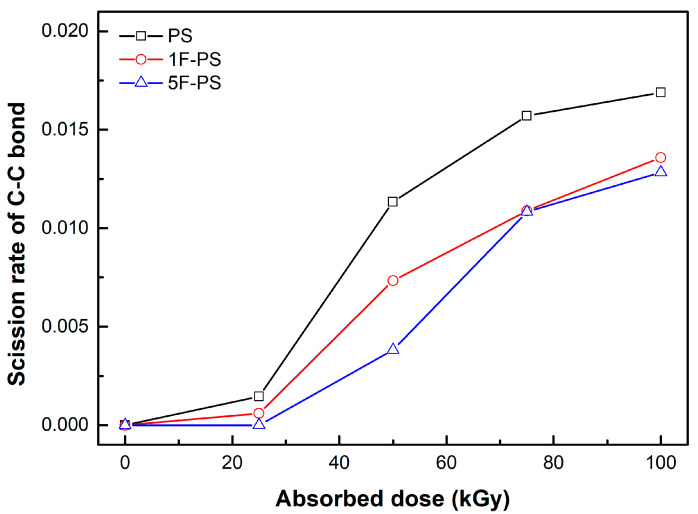
Scission rate of C-C bond of polymers.

**Figure 12:**
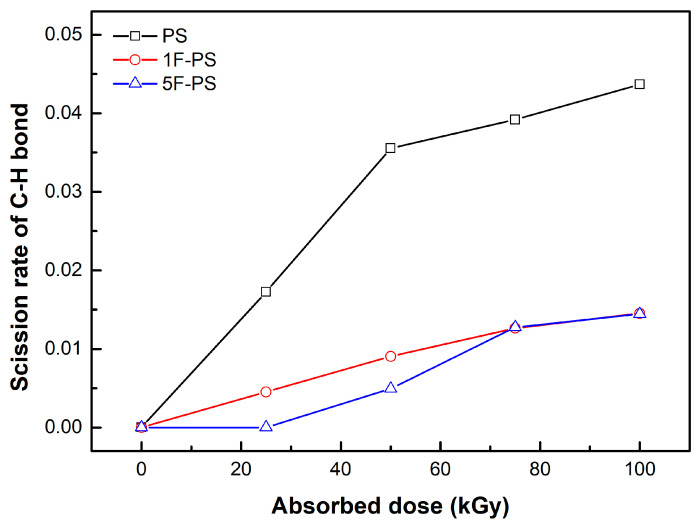
Scission rate of C-H bond of polymers.

**Figure 13:**
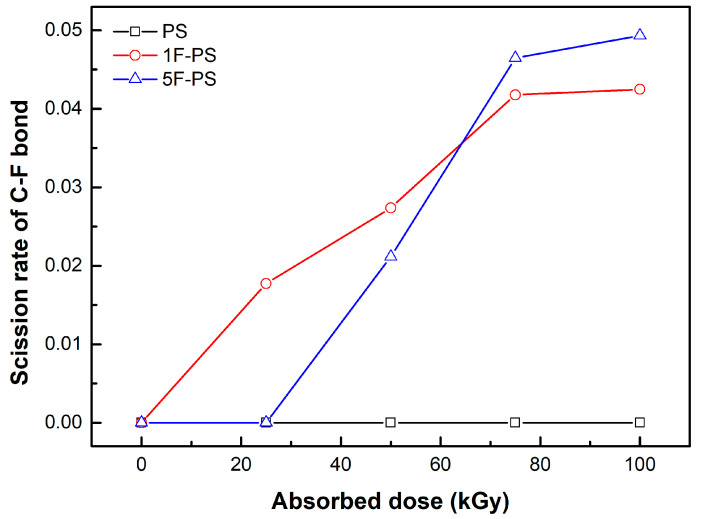
Scission rate of C-F bond of polymers.

In the process of the experiment (measuring average molecular weight by gel permeation chromatography after irradiation), both the scission and crosslinking processes had happened. The initial average molecular weights of polymers are 25,651.0, 27,468.3, 56,011.0 for PS, 1F-PS, and 5F-PS. The average molecular weight of PS increased to 28,129.0 at 100 kGy. In the case of polystyrene fluoride, the average molecular weight of 1F-PS increased to 29,691.5 at 50 kGy, decreased to 29,540.0 at 100 kGy, and the average molecular weight of 5F-PS increased to 57,630.0 at 25 kGy and decreased to 54,345.5 at 100 kGy. However, the scission process should be done before crosslinking process. So we assumed that the change of average molecular weight was dominant by the scission process. We normalized the difference of average molecular weight by matching the initial average molecular weight to zero, and then we calculated the absolute difference between the initial average molecular weight and after the irradiation as shown in Figure 10 [25,26,27]. We compared these differential values with the simulation results of the scission rate. Equations (4) and (5) are being applied for comparison.

(4) $$\alpha \;\left(scission\;rate\right)=\frac{N}{m}$$

(5) $$\;\delta \left(Ratio\;of\;Differential\;Mn\right)=\left(\frac{1}{M_{0}}-\frac{1}{Mn}\right)\times N$$

where *m* denotes the mass of bonds, *Mn* denotes the number average molecular weight after irradiation, *M*_0_ denotes the initial average molecular weight, and *N* is the differential number: the ratio between the average molecular weight of atoms (experiment results) and atom number (simulation value) after irradiation. The scission rates of the bonds which were simulated in the polymers are shown in Figure 11, Figure 12 and Figure 13.

The scission rate of the C-C bond was the highest in the polystyrene. The value of the scission rate was increased sharply in the region of 25 to 50 kGy for polystyrene and 1F-PS and 50 to 75 kGy for 5F-PS. These results were similar with the maximum dissociation rate region, which were 3 (#/kGy) from 25 to 50 kGy at the polystyrene, 1.96 (#/kGy) from 25 to 50 kGy at the 1F-PS, and 2.84 (#/kGy) from 50 to 75 kGy at the 5F-PS.

This relation was also investigated in the gel permeation chromatography results. The difference of Mn was the highest in the region of 25 to 50 kGy for polystyrene and 1F-PS. However, it is different in the 5F-PS case. This means that the dominant scission happened in the C-C bond for polystyrene and 1F-PS.

The scission rate of the C-H bond was also the highest in the polystyrene. The value of the scission rate was increased linearly in the region of 0 to 50 kGy for polystyrene and 1F-PS and 75 to 100 kGy for 5F-PS. The increment of the scission rate was decreased after 50 kGy for polystyrene and 75 kGy for 1F-PS, 5F-PS.

The scission rate of the C-F bond in the 1F-PS was higher than 5F-PS until 75 kGy. The scission process did not happen until 25 kGy in the 5F-PS. The number of C-F bonds in 5F-PS is 5 times larger than 1F-PS. However, the scission rate of C-F bonds in 5F-PS was similar to 1F-PS.

The total scission rate of PS was higher than 1F-PS, 5F-PS in the simulation. The results of the scission trend of PS, 1F-PS was similar with the measured value, particularly until 50 kGy. The measured value was decreased after 50 kGy.

**Figure 14:**
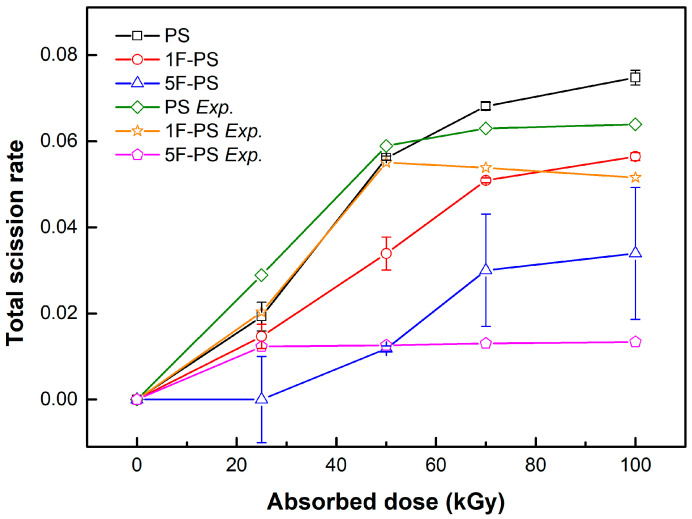
Total Scission Rate.

In the case of 5F-PS simulation, the scission did not happen until 25 kGy in the simulation. However, the measured value was increased until 25 kGy, and the rate of increase was the minimum in the data. The simulation results were compared with the experimental results and shown in Figure 14.

## 5. Discussion

The radiation enhancement effect with fluorine in the polystyrene has been determined by atomistic simulations to predict the possibility of using this method for modeling and simulation, which is irradiation reactions that occur in these materials.

The fluorinated PS backbone is made up of carbon–carbon bonds and the pendant groups are carbon-fluorine bonds. Both are extremely strong bonds. The basic properties of fluoropolymers from these two very strong chemical bonds. The quantity of the fluorine atom can be affected the crystallinity of PS and continuous covering around the carbon–carbon bonds and protect them from chemical attack, thus imparting chemical resistance and stability to the molecule.

In these polymers, chain scission (scission of C-C) was the primary reaction. The number of dissociation was increased proportionally with the absorbed dose. The ratio of increment of atoms in the simulation was the same with the measured (Mn) value.

5F-PS was found to be more resistant to radiation damage than 1F-PS and polystyrene. The benzene ring can be considered to act as a very efficient trap for F atoms due to the so-called cage effect. So, the F atoms are formed in an efficient cage of benzene molecules; the reactions (6) and (8) can predominate over reaction (9).

(6) $$\sim C_{6}F_{5}\;\to \;\sim C_{6}F_{4}•+\;F•$$

(7) $$\sim C_{6}F_{5}+\;F•\;\to \;\sim C_{6}F_{6}•$$

(8) $$R•+\sim C_{6}F_{5}\;\to {\;\mathrm{RC}}_{6}F_{5}•,\;\left[R•=\sim C_{6}F_{4}•,\;\sim C_{6}F_{5}•,\;\sim C_{6}F_{5}C_{6}F_{4}•\right]$$

(9) R•+ R• → RR

(10) R•+ F• → RF

(11) $$F•+\;F•\;\to {\;F}_{2}$$

The simulation results were reasonable especially calculating the scission ratio, and predicting the radiation resistance of aromatic fluorocarbon. In the simulation, we assumed that the absorbed dose of polystyrene and fluorinated polystyrene relatively increased cascade area 1.6, 2.3, 2.8, and 3.2 Å. In this assumption, we converted the whole energy of γ-ray to the kinetic energy of atoms which were participated in the cascade. We expected that the experiment results were different from the simulation results because of the difference between the average molecular weight of atoms (experiment results) and atom number (simulation value). However, we coupled the simulation results with the scission rate and differential ratio of Mn (the number of molecular weight) and figured out that the trend of scission rate was similar in the results comparing the simulation data and experimental data. In this study, the scission process was only considered to predict the effect of fluorine. We will update this method with the crosslinking process to overcome the unforeseeable circumstances—prediction of radiation enhancement before the irradiation.

## 6. Conclusions

Generally, the high energy of the gamma-rays and the formation of radicals led to the chain scissioning, resulting in a decrease in molecular weight of the polymer. The produced alkyl free radicals react with oxygen to form peroxyl free radicals through the abstraction of hydrogen led to the cleavage of the C-H, C-C, and C-F chains.

When aromatic fluorinated polymers are irradiated, the observed yield of breaks obtained by splitting C-F and C-C bonds is often lower than those from the C-H and C-C bonds in aliphatic hydrocarbon polymers. Namely, the fluorine atoms are not readily abstracted from fluorinated polymer by general molecules and radicals such as H, CH_3_, CF_3_, and C_2_F_5_. This is because of radiation resistance due to the combination of C-F binding force and aromatic resonance structure. Actually, the aromatic hydrocarbon biphenyl and terphenyl are reported to be sufficiently resistant to radiation, the fluorine atoms of aromatic fluorocarbon (i.e., 1F-PS and 5F-PS) are also expected to be resistant to radiation. The more fluorine content in the benzene, the highly ordered π-stacking of fluorinated benzene increases charge carrier mobilities, suggesting stabilization against bond rupture by the radiation. So, it seems reasonable to conclude that the fluorine will be hardly abstracted from 5-F rather than 1-F based on simulation and measurement results [28].

## Figures and Tables

**Table 1:** Maximum dissociation number of each bond in polystyrene @ 1 ps.

PKA Radius (Å)	Absorbed Dose (kGy)	No. of Simulations	Maximum Dissociation Number of C-C Bond	Maximum Dissociation Number of C-H Bond	Total Dissociation Number	Dissociation Rate (#/kGy)
1.6	25	10	7	35	42	1.68
2.3	50	10	50	71	117	3
2.8	75	10	67	78	140	0.92
3.2	100	10	70	87	157	0.68

**Table 2:** Maximum dissociation number of each bond in 1F-PS (poly(4-fluorostyrene)) @ 1 ps.

PKA Radius (Å)	Absorbed Dose (kGy)	No. of Simulations	Maximum Dissociation Number of C-C Bond	Maximum Dissociation Number of C-F Bond	Maximum Dissociation Number of C-H Bond	Total Dissociation Number	Dissociation Rate (#/kGy)
1.6	25	10	3	3	30	36	1.44
2.3	50	10	31	6	43	85	1.96
2.8	75	10	46	8	73	127	1.68
3.2	100	10	57	9	74	140	0.52

**Table 3:** Maximum dissociation number of each bond in 5F-PS (poly(2,3,4,5,6-pentafluorostyrene)) @ 1 ps.

PKA Radius (Å)	Absorbed Dose (kGy)	No. of Simulations	Maximum Dissociation Number of C-C Bond	Maximum Dissociation Number of C-F Bond	Maximum Dissociation Number of C-H Bond	Total Dissociation Number	Dissociation Rate (#/kGy)
1.6	25	10	0	0	0	0	0
2.3	50	10	16	15	15	47	1.88
2.8	75	10	45	38	35	118	2.84
3.2	100	10	53	43	37	133	0.6

**Table 4:** Maximum dissociation number of each bond in polystyrene @ 1ps.

Absorbed Dose (kGy)	Mn of PS	Mn of 1F-PS	Mn of 5F-PS	The Ratio of Differential Mn (PS)	The Ratio of Differential Mn (1F-PS)	The Ratio of Differential Mn (5F-PS)
0	25,651.0	27,468.3	56,011.0	0.00000	0.00000	0.00000
25	26,714.3	28,249.0	57,630.0	0.02887	0.02032	0.01229
50	27,917.6	29,691.5	54,441.0	0.05890	0.05505	0.01261
75	28,089.0	29,639.0	54,391.0	0.06296	0.05384	0.01303
100	28,129.0	29,540.0	54,345.5	0.06391	0.05156	0.01340

## Data Availability

Not applicable.
